# Correlation between Either *Cupriavidus* or *Porphyromonas* and Primary Pulmonary Tuberculosis Found by Analysing the Microbiota in Patients’ Bronchoalveolar Lavage Fluid

**DOI:** 10.1371/journal.pone.0124194

**Published:** 2015-05-22

**Authors:** Yuhua Zhou, Feishen Lin, Zelin Cui, Xiangrong Zhang, Chunmei Hu, Tian Shen, Chunyan Chen, Xia Zhang, Xiaokui Guo

**Affiliations:** 1 Department of Medical Microbiology and Parasitology, Institutes of Medical Sciences, Shanghai Jiao Tong University School of Medicine, Shanghai, China; 2 Department of Tuberculosis, Nanjing Chest Hospital, Nanjing, Jiangsu, China; 3 Department of Emergency Medicine, Rui Jin Hospital Shanghai Jiao Tong University School of Medicine, Shanghai, China; 4 Department of Preventive Medicine, Shanghai Jiao Tong University School of Medicine, Shanghai, China; Fundació Institut d’Investigació en Ciències de la Salut Germans Trias i Pujol. Universitat Autònoma de Barcelona. CIBERES, SPAIN

## Abstract

Pulmonary tuberculosis (TB) has gained attention in recent decades because of its rising incidence trend; simultaneously, increasing numbers of studies have identified the relationship between microbiota and chronic infectious diseases. In our work, we enrolled 32 patients with primary TB characterised by unilateral TB lesion formation diagnosed by chest radiographic exam. Bronchoalveolar lavage fluid was taken from both lungs. Twenty-four healthy people were chosen as controls. Pyrosequencing was performed on the V3 hypervariable region of 16S rDNA in all bacterial samples and used as a culture-independent method to describe the phylogenetic composition of the microbiota. Through pyrosequencing, 271,764 amplicons were detected in samples and analysed using tools in the Ribosomal Database Project (RDP) and bioinformatics. These analyses revealed significant differences in the microbiota in the lower respiratory tract (LRT) of TB patients compared with healthy controls; in contrast, the microbiota of intra/extra-TB lesions were similar. These results showed that the dominant bacterial genus in the LRT of TB patients was *Cupriavidus* and not *Streptococcus*, which resulted in a significant change in the microbiota in TB patients. The abundance of *Mycobacteria* and *Porphyromonas* significantly increased inside TB lesions when compared with non-lesion-containing contralateral lungs. From these data, it can be concluded that *Cupriavidus* plays an important role in TB’s secondary infection and that in addition to *Mycobacteria*, *Porphyromonas* may also be a co-factor in lesion formation. The mechanisms underlying this connection warrant further research.

## Introduction

Pulmonary tuberculosis (TB) is an ancient chronic infectious disease that has always been a crucial challenge for public health [1]. Clinically, typical TB presents as granuloma-like lesions in the lung and chronic consumptive symptoms [2,3]. In the field of TB research, the pathogen *Mycobacteria tuberculosis* is the focal point, and issues related to its virulence, pathogenicity and drug resistance have been extensively researched [4,5]. With the development of systematic biology, the concept and mechanisms underlying persistent infections has changed and which suggests that infectious diseases reflect an equilibrium between the host and the pathogen that is established and maintained by a broad network of interactions that occur across scales, ranging from molecular and cellular to whole organism and population levels [6,7]. Chronic TB is a unique infection in the human body that is different from other types of diffuse pulmonary infections, and the formation of granulomas isolates the pathogens involved from other lung tissue. However, when judged through the lens of new infection concepts, changes in the microbiota in the lungs likely play an important role in the pathological process [8].

The rapid development of nucleotide sequencing technology has made it possible to uncover the composition of microbiota in some cavities in the human body [9]. For example, many published articles have examined the bacterial flora in the vagina, intestine and lung, attempting to determine the relationship between changes in local microbiota and such diseases as obesity, inflammation or diabetes [10,11]. In our previous researches, we also described the difference among the microbiota from healthy individuals, patients with hospital acquired pneumonia and patients with community acquired pneumonia [12,13]. From the viewpoint of the current studies, the stability or instability of the microbiota *in vivo* deeply influences the maintenance of health and the onset or progression of disease [7]. We hypothesise that the understanding of the link between the microbiota and persistent infection in the lungs of TB patients is currently in the very early stages and is an important topic for further research; therefore, the composition of the microbiota in intra- or extra-TB lesion areas needs to be analysed.

Bronchoalveolar lavage (BAL) fluid, which is obtained by means of bronchoscopy, is the carrier that best reflects the composition of the microbiota in the lower respiratory tracts (LRT) and alveoli of humans [14,15]. In this study, we enrolled 32 primary TB patients with unilateral TB lesion formation, with the opposite lungs presenting as ‘healthy and lesion-free’ upon chest X-ray and Computed tomography (CT) examination. After the patients had been hospitalised and received a standard 2-week-long anti-TB treatment, bronchoalveolar lavage samples were collected. Alveolar lavage fluid was taken from both the lung with the TB lesion and the contralateral normal pulmonary tissue. Twenty-four healthy volunteers of similar ages were also selected, and their respiratory tract secretions were collected as normal controls.

A common method in microecology is based on massively parallel pyrosequencing of bacterial 16s rDNA amplicons in the V3 region; the feasibility of this approach has been shown in other infections [16]. Our objectives were to analyse and compare the microbiota of the TB lesion-forming area of the lung with that of the non-lesion forming areas of the lungs, and to compare it with the microbiota of TB patients and with that of healthy individuals. Our comparative study of these different populations offers some primitive conclusions and insights of a breadth previously unavailable in the study of pulmonary tuberculosis.

## Materials and Methods

### Ethics Statement

Bronchoscopy is a common method used in the field of diagnosis and therapy of the tuberculosis, especially in endobronchial tuberculosis. Its merits and safety were proven in previous clinical operations [17]. The study was approved by the ethics committee of Nanjing Chest Hospital. All the clinical works fulfilled the guideline of the bronchoscopy treatment towards the pulmonary tuberculosis [18,19]. As a kind of invasive operation, bronchoscopy requires strictly trained operator and the formal consent from every patient before being undergone. In clinic, every enrolled patient must sign a notification which declaims the aims, methods, merits and risks of this operation in detail. On the base of patients’ full understanding and when the signatures were obtained, the bronchoscopy was allowed to be undergone and the bronchoalveolar lavage fluid was obtained. If the bronchoalveolar lavage fluid was suit for our research work, the corresponding patient would be further informed that his/her sample would be further used in research work after the relative clinical works were done. In this step, after obtaining the verbal consent from the patient, our research would be undertaken. Our lab work was started after the samples being discarded. This study didn't involve patient private information and any adjustment or difference in clinical treatment. Therefore, all the samples were not intended to be collected specially for the research but a step after the routine clinical exam. We provided the scanning file of the pre-operation notification (original manuscript in Chinese used in clinical work) and the translated file made by authorized translator (manuscript in English) in supporting information (S1 and S2 Files).

The volunteers as normal control were acted by the relatives of the members of our work group. All of them agreed to support our research work. Unlike the patients’ samples, the samples of volunteers were saliva which collected by non-invasive method. After inquiring the ethics committee of Nanjing Chest Hospital, they agreed that we could require them to provide their verbal consents. Of cause, they were informed all the details of the usage of their samples.

### Patient selection and research design

Thirty-two patients between the ages of 18 to 48 years old with primary tuberculosis were enrolled in this study. The average age was 30.4 and median age of 29. Only 3 patients had smoking histories. The ratio of males to females in the study cohort was 21/11 (Table 1). All of the patients were clinically diagnosed with primary pulmonary tuberculosis via sputum stain, tuberculin PPD (purified protein derivative) test, blood TB markers and chest radiographic exam. Chest radiography was the most important criteria for patient inclusion in the study because it confirmed single-sided lung TB lesion formation as judged by X-ray or CT exam; this criteria ensured that every patient had one side on the contralateral lung that was normal according to a chest imaging exam [20]. None of the patients had other basic lung diseases or systemic or immune-compromising diseases, such as diabetes or HIV. In addition, none of the patients had received antibiotics for at least 3 months before sample collection.

**Table 1:** Clinical information of the enrolled TB patients.

No	Gender	Age	Smoking habit	Family history	X-ray Diagnosis*	Sputum Stain	PPD	Blood TB markers
1	F	43	N	N	(-)/L	1+	N	LAM+
2	M	48	N	N	U/(-)	N	P	LAM+
3	F	35	N	N	(-)/L	2+	P	TB-ab+
4	F	49	N	N	(-)/L	1+	P	(-)
5	M	32	N	N	U,L/(-)	3+,4+	P	Tb-Ab(+),LAM(+)
6	M	43	400/Y	N	U/(-)	1+	P	Tb-Ab+,LAM+,38KDa+
7	M	31	400/Y	N	(-)/U	N	P	(-)
8	M	22	N	N	(-)/U,M,L	N	P	Tb-Ab+
9	M	22	N	N	U/(-)	N	P	Tb-Ab+
10	M	19	N	N	(-)/U	N	P	(-)
11	M	30	N	N	(-)/U,M,L	1+,4+	P	Tb-ab+,LAM+,38KDa+
12	F	27	N	N	(-)/U	1+	P	Tb-Ab+
13	M	48	N	N	U/(-)	N	N	(-)
14	M	34	N	N	U/(-)	4+	P	(-)
15	M	47	N	N	U/(-)	1+	P	Tb-Ab+
16	M	20	N	N	L/(-)	N	P	Tb-ab+,LAM+,38KDa+
17	M	36	N	N	U/(-)	N	P	Tb-ab+,LAM+,38KDa+
18	M	25	N	N	(-)/U	1+	P	Tb-Ab+
19	F	28	N	N	U/(-)	2+	P	Tb-ab+,LAM+,38KDa+
20	F	30	N	N	U/(-)	2+	P	Tb-Ab+-
21	F	28	N	N	(-)/U	1+	P	Tb-Ab+,LAM+,38KDa+
22	F	18	N	N	U/(-)	1+	P	Tb-Ab+
23	M	18	N	N	(-)/U,M	4+	N	Tb-ab+,LAM+,38KDa+
24	F	23	N	N	(-)/L	1+	P	Tb-Ab+
25	F	23	N	N	U/(-)	N	P	LAM+
26	M	36	N	N	U/(-)	N	P	(-)
27	M	18	N	N	(-)/L	1+	P	LAM+,38KDa+
28	M	39	200/Y	N	(-)/U	1+	P	(-)
29	M	32	N	N	(-)/U	N	P	(-)
30	F	24	N	N	M/(-)	1+	N	Tb-ab+,LAM+,38KDa+
31	M	25	N	N	U/(-)	1+	P	(-)
32	M	21	N	N	(-)/L	N	P	TB-Ab+,LAM+

*In the column of X-ray diagnosis shows the location of TB lesion in each patient. Left side of the ‘/’ refers to the left lung and right side refers to right lung. U, M, L refer to the upper, middle and lower lobi of lungs respectively and ‘-’ refers to lesion-free lungs.

Before the undertaking of bronchoscopy, every patient accepted a standard two-week-long anti-TB treatment with a combination of isoniazid, rifampicin, ethambutol and pyrazinamide and thus aimed to confine the activation of pathogens and reduce the risk of its dissemination *in vivo*. On the other hand, it would help to form the localized TB lesion in lung. All above were according to the treating guideline towards the primary TB and endobronchial tuberculosis [18]. After these treatments, chest radiographic exams were retaken to determine pulmonary lesion status and position. Then, bronchoalveolar lavage guided by bronchofibroscopy was undertaken. For each patient, two lavage samples were collected: one from the normal side (defined as Group A) and one from the opposite, lesion-forming lung area (defined as Group B). On the assumption that the side with TB lesion had a higher abundance of pathogens than the opposite side, we undertook lavage in the lesion-free side prior to the lesion-bearing side. When the lavage in the lesion-free side was done, the bronchoscopy was sterilized and then the operation would be continued. Although, the multi-contamination could not be fully avoided because of the limit of the instrument and operating skills, for example, the trachea acted as the common pathway when push-in and pull-out the bronchoscopy, we believe that these measures would minimize the bias by present methodology. After the operation, the anti-TB medicine treatment would be continued after the operation until the end of required medicine course of treatment by guideline.

Twenty-four healthy people were also selected as normal controls. All of the controls lived in the same region as the patients and had similar lifestyle and eating habits. They had no smoking history, basic pulmonary disease, severe oral disorders, systemic diseases or other diseases known to affect the lungs’ microbiota. The samples from the healthy controls were a mixture of saliva and pharyngeal secretions obtained by deep coughing in the early morning before gargling. These samples were defined as Group H.

### Establishment of the 16S rDNA library and 454 pyrosequencing

The protocols for DNA extraction from samples and PCR enrichment of the V3 region of 16S have been previously described [12]. Because the lavage fluid from TB patients is highly infectious, pre-extraction sample preparation was modified as follows: all lavage samples were heated and pasteurised at 80°C for 2 hours and then centrifuged at 12,000 x *g* for 10 minutes. The supernatant was discarded, and the sediment was collected. The remaining sediment was resuspended in sterile saline, and DNA extraction protocols were performed.

PCR enrichment of the V3 hyper-variable region of 16S rRNA was performed with forward primer 5’-XXXXXXXX-TACGGGAGGCAGCAG-3’ and reverse primer 5’-XXXXXXXX-ATTACCGCGGCTGCTGG-3’. The 5’ termini of each primer contained a different 8-base oligonucleotide tag before the hyphen, while the sequence after the hyphen was able to pair with the sequence at the V3 end region.

To ensure that there was sufficient PCR product to harvest, a two-step PCR strategy was used. The first step was conducted in a 25-ul reaction volume containing 2.5 μl of PCR buffer (TaKaRa), 0.625 U ExTaq (TaKaRa), 0.1 μl of BSA (TaKaRa), and 2 μl of primer solution with 100 μmol of each forward and reverse primer. Fifty nanograms of extracted DNA were added as a template. ddH_2_O was added to reach the final reaction volume. The touchdown PCR conditions were as follows: 5 min at 94°C for initial denaturation, 1 min at 94°C for denaturation, 1 min at 65°C for annealing and 1 minute at 72°C for extension, with the annealing temperature decreasing by 0.5°C for each step of the 20 cycles. The reaction volume in the second step of the PCR was 50 μl. Five microliters of the reaction from Step One were used as a template for this reaction with 5 μl of PCR buffer (TaKaRa), 1.25 U ExTaq (TaKaRa), 0.2 μl of BSA (TaKaRa), and 24 μl of water with 200 μmol of each barcoded forward and reverse primer. The heating cycle lasted 1 minute at 94°C for denaturation, 1 minute at 55°C for annealing, and 1 minute at 72°C for extension for five cycles with the temperature kept at 20°C after the reaction. The sequencing work was performed at the Chinese National Human Genome Centre in Shanghai using a Roche 454 FLX sequencing machine. The sequences were stored as SRA accession numbers SRA051957 and SRA054227.

### Data analysis by bio-informatics and statistical methods

The datasets were taxonomically grouped using the Ribosomal Database Project (RDP) Naive Bayesian classifier with a confidence level of 90% [21]. Gross sequencing data were first searched for the linker, primers, and their reverse complements using the platform provided by the centre. Identified primer sequences were trimmed from each sequence read. Sequence reads that did not contain the 5’-end primer were removed from the data set. The same program was also used for barcode identification. Barcodes were identified within the first 25 bases of the reads. Sequence reads were binned into FASTA files based on the different barcodes.

Individual sequences were aligned using Aligner tools, and aligned sequence files for each sample were processed using complete-linkage clustering with distance criteria. We used the Uclust algorithm to cluster all of the sequences with a cut-off value of 97%; after clustering, we used the representative sequence of each type as the operational taxonomic unit (OTU) and recorded each OTU sequence representing the number of sequences and the classification information. These data were used to calculate the Shannon diversity and evenness indices. The Fast UniFrac program was used to analyse phylogenetic microbial communities from two types of samples [22]. A heatmap was drawn using R language. Shannon diversity was estimated using Estimate S Win 8.20 software.

Although the bio-informatics methods were able to provide us a phylogenetic distance or relationships among samples, we could only describe the difference in the whole-body level. In order to find out which genera played the most important roles in the microbiota’s change and which genera varied obviously in ratios when comparing the samples from lesion-bearing and lesion-free lobi, we used a paired Wilcoxon signed-rank test by SPSS (Statistical Package for the Social Sciences) 19.0 to compare the percentages of genera which were detected simultaneously in both sides of the patients’ lungs.

### Data availability statement

We have uploaded our 16s V3 rDNA sequences obtained by 454 platform in the public repository named SRA (Sequence Read Archive, http://www.ncbi.nlm.nih.gov/Traces/sra/). The accession number of the sequences from healthy individuals is SRR493275. The accession numbers of the sequences from TB patients are SRS375896, SRX204970 and SRR617950. All these data are in the state of “public”. Meanwhile, according to the PLOS data policy, we also deposited our raw data in FASTA file format to the integrated repository named DRYAD (http://datadryad.org/) and all files are available by the DOI: 10.5061/dryad.mt24h.

## Results

**Fig 1:**
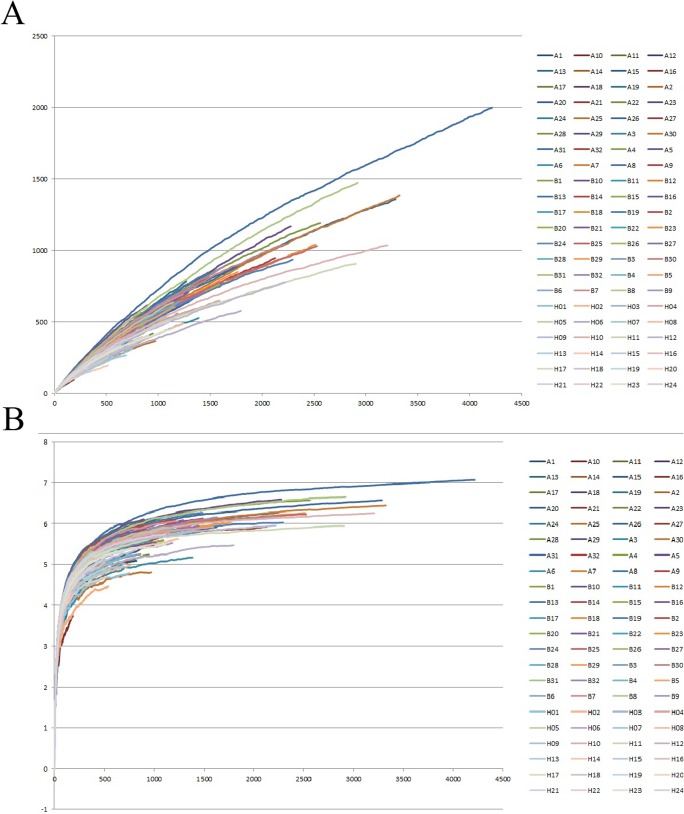
Rarefaction and Shannon Weaver index analysis were used to evaluate the richness and diversity of amplicons. (A) Rarefaction curves were used to estimate the richness of the amplicons in each sample. (B) Shannon-Weaver index curves were used to estimate the amplicons’ diversity of each sample.

A total of 271,164 PCR amplicons were detected by pyrosequencing in all samples that were approximately 200 bp in length, and each sample contained 3081.4 amplicons on average. The diversity and richness of the amplicons in each sample were evaluated using Shannon-Weaver index and rarefaction curves, which are shown as Fig 1. The mean Shannon diversity indices were 5.40, 5.71, and 5.31 for Groups A, B, and H respectively. The patient indices in Group B were significantly higher than those of Group H (p = 0.004). Nearly all of the Shannon curves featured two-component curves that included a sharp slope in the beginning and a less-sharp slope for the remainder.

**Fig 2:**
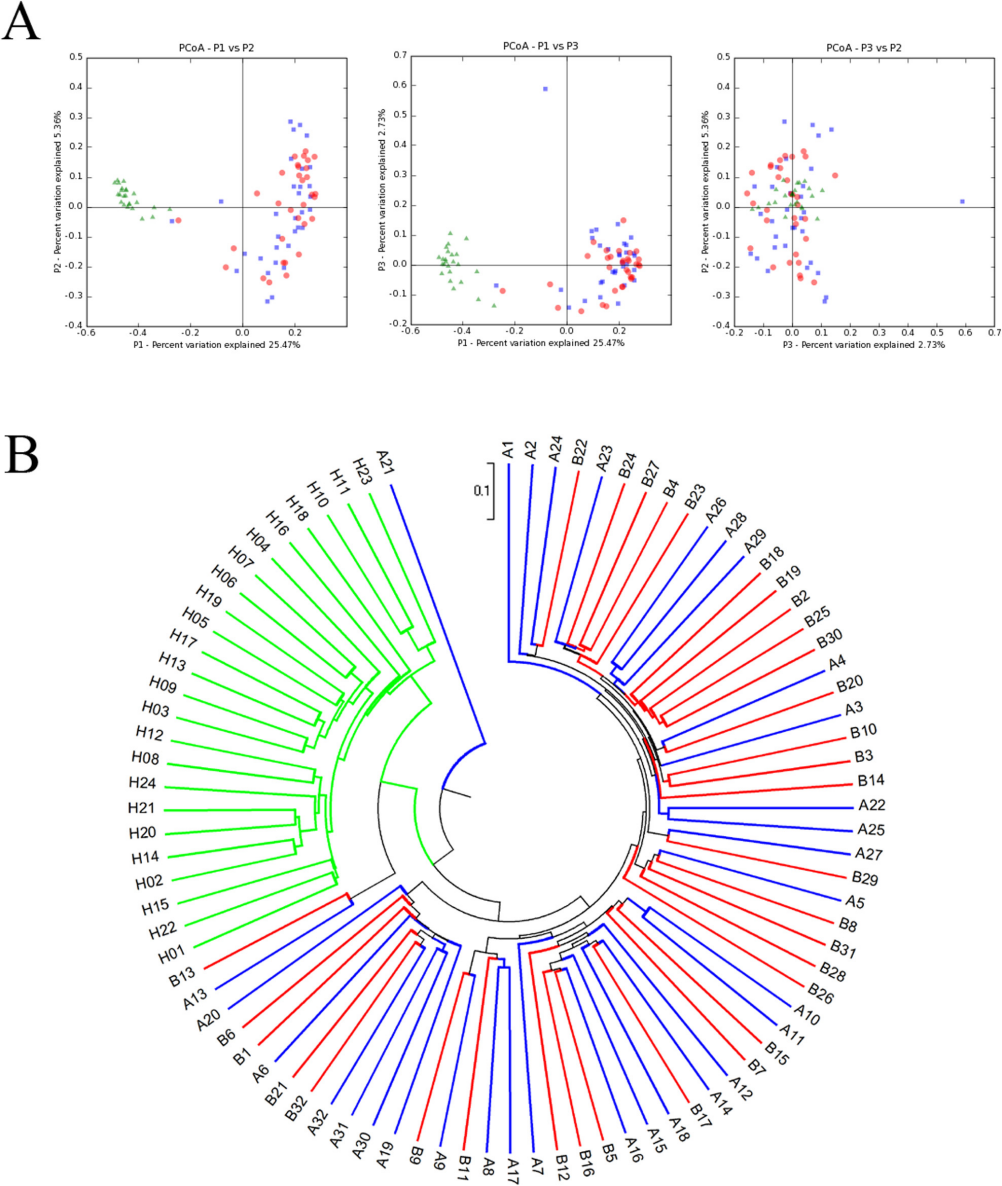
Principal component analysis (PCoA) and phylogenetic trees between the samples. (A) PCoA shows the relationship between the samples. Each data point represents the microbiome identified from one sample. The green, blue and red points represent samples from Groups H, A and B, respectively. (B) A phylogenetic tree shows the phylogenetic distance between samples. The green, blue and red points represent samples from Groups H, A and B, respectively.

The similarities between the microbiota of selected individuals are shown in Fig 2, using principal coordinate analysis (PCoA) and a phylogenetic tree. After pyrosequencing, all detected amplicons were clustered in Uclust with a cutoff threshold of 97%, followed by the generation of 6319 OTUs. The phylogenetic relationships between the microbiota from different groups became visible after calculating the Unifrac distance, as shown in Fig 2. Fig 2A demonstrates that the microbiota from Group H were phylogenically distant from the microbiota of TB patients, and Fig 2B shows that the microbiota from these two groups clearly fell into two different sub-branches. In contrast, the microbiota from Groups A and B were too similar to clearly separate.

**Fig 3:**
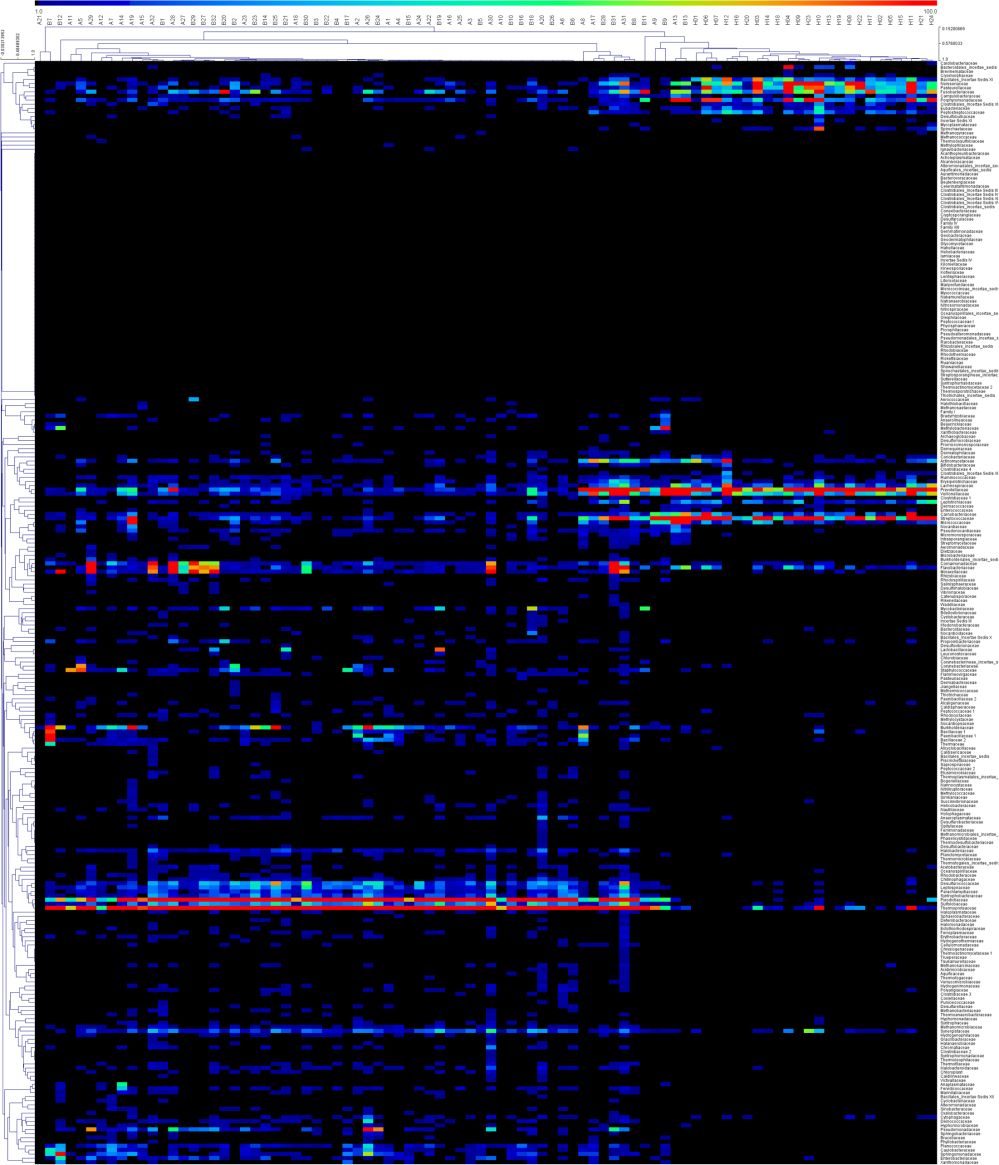
Hierarchical clustering heatmap of the microbiota of samples. Bacterial family names are listed on the right side of the heatmap, and the sample names are listed on the top. Dendrograms at the top and left of the map indicate the phylogenetic relationship between the samples and families; the intensity of the cells’ colour represents the abundance of the amplicons belonging to each family and sample.

To describe the detailed composition features of the microbiota in detail, amplicons were sorted into bacterial families. The hierarchical clustering heatmap shown in Fig 3 displays the abundance of each family and the relationships between families and samples. Group H is clustered at the right side of the figure, and patients (Groups A and B) are clustered to the left. The distribution of hot points clearly suggests significant differences between the microbiota of healthy controls and that of TB patients. When all amplicons were sorted to the level of bacterial genera, amplicons belonging to every detected genus were counted. The top 30 genera with the highest percentage in each group are presented in Table 2. These results showed that *Streptococcus* was the most abundant genus in healthy specimens, and *Cupriavidus* was the dominant genus in patients. The percentages of genera from both sides were compared using a Wilcoxon signed-rank test for TB patients. This analysis revealed that with the exception of *Mycobacteria* and *Porphyromonas*, whose percentages were much higher in Group B, the detected genera did not differ significantly among TB patients (Groups A and B; Table 3).

**Table 2:** Top 30 genera in both lungs in TB patients and in healthy people.

R	Health		lung without lesion		lung with lesion	
	Genera	Mean(%)	Genera	Mean (%)	Genera	Mean (%)
1	*Streptococcus*	21.67	*Cupriavidus*	5.13	*Cupriavidus*	4.59
2	*Prevotella*	10.11	*Prevotella*	4.24	*Prevotella*	4.50
3	*Haemophilus*	4.00	*Acinetobacter*	3.03	*Acinetobacter*	3.67
4	*Veillonella*	3.62	*Streptococcus*	3.00	*Streptococcus*	3.29
5	*Fusobacterium*	3.39	*Staphylococcus*	2.81	*Fusobacterium*	2.84
6	*Capnocytophaga*	3.24	*Fusobacterium*	2.64	*Rummeliibacillus*	1.57
7	*Granulicatella*	3.14	*Pseudomonas*	1.87	*Flavobacterium*	1.55
8	*Gemella*	2.81	*Flavobacterium*	1.58	*Pseudomonas*	1.55
9	*Neisseria*	1.78	*Veillonella*	1.47	*Veillonella*	1.51
10	*Leptotrichia*	1.75	*Rothia*	1.44	*Staphylococcus*	1.28
11	*Actinomyces*	1.47	*Rummeliibacillus*	1.33	*Mycobacterium*	1.05
12	*Rothia*	1.04	*Comamonas*	0.98	*Actinomyces*	0.97
13	*Aggregatibacter*	0.87	*Actinomyces*	0.85	*Comamonas*	0.85
14	*Campylobacter*	0.80	*Sphingomonas*	0.79	*Lactobacillus*	0.85
15	*Porphyromonas*	0.72	*Brevibacillus*	0.72	*Escherichia/Shigella*	0.81
16	*Selenomonas*	0.72	*Granulicatella*	0.69	*Methylobacterium*	0.78
17	*Peptostreptococcus*	0.66	*Escherichia/Shigella*	0.61	*Granulicatella*	0.63
18	*Parvimonas*	0.54	*Deinococcus*	0.47	*Rothia*	0.58
19	*Treponema*	0.49	*Bosea*	0.45	*Sphingomonas*	0.46
20	*Cardiobacterium*	0.18	*Thermus*	0.45	*Brevibacillus*	0.44
21	*Atopobium*	0.18	*Mycobacterium*	0.42	*Neisseria*	0.34
22	*Tannerella*	0.06	*Corynebacterium*	0.41	*Capnocytophaga*	0.34
23	*Mycoplasma*	0.06	*Haemophilus*	0.34	*Haemophilus*	0.32
24	*Peptococcus*	0.04	*Gemella*	0.33	*Leptotrichia*	0.30
25	*Kingella*	0.04	*Propionibacterium*	0.30	*Gemella*	0.29
26	*Oribacterium*	0.04	*Solobacterium*	0.30	*Deinococcus*	0.27
27	*Dialister*	0.04	*Neisseria*	0.30	*Corynebacterium*	0.27
28	*Megasphaera*	0.03	*Pelomonas*	0.26	*Propionibacterium*	0.27
29	*Mogibacterium*	0.03	*Capnocytophaga*	0.24	*Pelomonas*	0.25
30	*Corynebacterium*	0.02	*Thauera*	0.23	*Porphyromonas*	0.22

**Table 3:** Paired Wilcoxon signed rank test comparison of the abundences between the genera in TB patients’ lungs with lesions and lungs without lesions.

Genera	Z	Asymp. Sig. (2-tailed)
*Mycobacterium*	-2.321	0.020
*Porphyromonas*	-2.240	0.025
*Azospirillum*	-1.826	0.068
*Bosea*	-1.820	0.069
*Thermus*	-1.789	0.074
*Rothia*	-1.714	0.086
*Sphingomonas*	-1.613	0.107
*Aquabacterium*	-1.604	0.109
*Methylophilus*	-1.604	0.109
*Parvimonas*	-1.604	0.109
*Sphingobacterium*	-1.604	0.109
*Lactobacillus*	-1.572	0.116
*Turicibacter*	-1.483	0.138
*Weissella*	-1.483	0.138
*Eubacterium*	-1.461	0.144
*Hyphomicrobium*	-1.461	0.144
*Stenotrophomonas*	-1.461	0.144
*Delftia*	-1.376	0.169
*Aggregatibacter*	-1.342	0.180
*Anaerococcus*	-1.342	0.180
*Cloacibacterium*	-1.342	0.180
*Erysipelotrichaceae_incertae_sedis*	-1.342	0.180
*GpI*	-1.342	0.180
*Mesorhizobium*	-1.342	0.180
*Novosphingobium*	-1.342	0.180
*Serratia*	-1.342	0.180
*Shuttleworthia*	-1.342	0.180
*Thauera*	-1.342	0.180
*Methylobacterium*	-1.334	0.182
*Propionibacterium*	-1.303	0.192
*Anoxybacillus*	-1.214	0.225
*Dyadobacter*	-1.183	0.237
*Acidovorax*	-1.153	0.249
*Streptophyta*	-1.153	0.249
*Staphylococcus*	-1.130	0.258
*Acinetobacter*	-1.121	0.262
*Exiguobacterium*	-1.120	0.263
*Pseudoxanthomonas*	-1.095	0.273
*Roseomonas*	-1.095	0.273
*Pseudomonas*	-1.093	0.274
*SR1_genera_incertae_sedis*	-1.070	0.285
*Bulleidia*	-1.069	0.285
*Mycoplasma*	-1.069	0.285
*Leptotrichia*	-1.067	0.286
*Capnocytophaga*	-1.022	0.307
*Granulicatella*	-1.003	0.316
*Aeribacillus*	-1.000	0.317
*Aeromicrobium*	-1.000	0.317
*Alkaliphilus*	-1.000	0.317
*Alloscardovia*	-1.000	0.317
*Arcobacter*	-1.000	0.317
*Atopococcus*	-1.000	0.317
*Cardiobacterium*	-1.000	0.317
*Catonella*	-1.000	0.317
*Desulfomicrobium*	-1.000	0.317
*Devosia*	-1.000	0.317
*Epilithonimonas*	-1.000	0.317
*Gordonia*	-1.000	0.317
*Gp4*	-1.000	0.317
*Hydrogenophilus*	-1.000	0.317
*Hymenobacter*	-1.000	0.317
*Kingella*	-1.000	0.317
*Klebsiella*	-1.000	0.317
*Limnobacter*	-1.000	0.317
*Luteibacter*	-1.000	0.317
*Marmoricola*	-1.000	0.317
*Meiothermus*	-1.000	0.317
*Methyloversatilis*	-1.000	0.317
*Moraxella*	-1.000	0.317
*Murdochiella*	-1.000	0.317
*Nesterenkonia*	-1.000	0.317
*Nubsella*	-1.000	0.317
*Oxalicibacterium*	-1.000	0.317
*Peptostreptococcaceae_incertae_sedis*	-1.000	0.317
*Peredibacter*	-1.000	0.317
*Pontibacillus*	-1.000	0.317
*Psychrobacter*	-1.000	0.317
*Tsukamurella*	-1.000	0.317
*Yaniella*	-1.000	0.317
*Clostridium XlVa*	-0.978	0.328
*Atopostipes*	-0.968	0.333
*Brevibacillus*	-0.931	0.352
*Lactococcus*	-0.921	0.357
*Streptococcus*	-0.901	0.367
*Haemophilus*	-0.876	0.381
*Enhydrobacter*	-0.784	0.433
*Deinococcus*	-0.776	0.438
*Acetobacter*	-0.734	0.463
*Erythrobacter*	-0.734	0.463
*Dialister*	-0.730	0.465
*Paracoccus*	-0.730	0.465
*Rhizobium*	-0.730	0.465
*Selenomonas*	-0.730	0.465
*TM7_genera_incertae_sedis*	-0.684	0.494
*Scardovia*	-0.674	0.500
*Phenylobacterium*	-0.663	0.508
*Ralstonia*	-0.652	0.515
*Cupriavidus*	-0.636	0.525
*Bifidobacterium*	-0.535	0.593
*Brevundimonas*	-0.535	0.593
*Finegoldia*	-0.535	0.593
*Massilia*	-0.535	0.593
*Raoultella*	-0.535	0.593
*Actinomyces*	-0.495	0.620
*Treponema*	-0.459	0.646
*Anaeroglobus*	-0.447	0.655
*Mobiluncus*	-0.447	0.655
*Niabella*	-0.447	0.655
*Novispirillum*	-0.447	0.655
*Parasegetibacter*	-0.447	0.655
*Roseburia*	-0.447	0.655
*Sphingopyxis*	-0.447	0.655
*Zoogloea*	-0.447	0.655
*Veillonella*	-0.433	0.665
*Peptostreptococcus*	-0.420	0.674
*Enterococcus*	-0.365	0.715
*Jeotgalicoccus*	-0.365	0.715
*Chryseobacterium*	-0.356	0.722
*Campylobacter*	-0.345	0.730
*Prevotella*	-0.299	0.765
*Flavobacterium*	-0.296	0.767
*Escherichia/Shigella*	-0.274	0.784
*Gemella*	-0.267	0.790
*Megasphaera*	-0.255	0.799
*Neisseria*	-0.245	0.807
*Pelomonas*	-0.226	0.821
*Fusobacterium*	-0.216	0.829
*Solobacterium*	-0.196	0.845
*Rummeliibacillus*	-0.196	0.845
*Corynebacterium*	-0.178	0.859
*Comamonas*	-0.175	0.861
*Nocardioides*	-0.169	0.866
*Bacillus*	-0.157	0.875
*Alcanivorax*	-0.135	0.893
*Pedobacter*	-0.135	0.893
*Pedomicrobium*	-0.135	0.893
*Aeromonas*	-0.105	0.917
*Atopobium*	-0.105	0.917
*Bradyrhizobium*	0.000	1.000
*Mogibacterium*	0.000	1.000
*Salmonella*	0.000	1.000
*Soonwooa*	0.000	1.000

The rates of positive detection of *Mycobacteria* were also evaluated using this statistical method. As Table 4 shows, a fourfold table chi-square test detected no significant differences in the positive rate between the clinical sputum stain method and pyrosequencing. Table 5 compares the positive rate of Group A to that of Group B and indicates that the probability of *Mycobacteria* detection in both sides by pyrosequencing was quite similar.

**Table 4:** Comparison between sputum stain and PYRO for detecting mycobacteria.

		Sputum		Total
		+	-	
**PYRO**	**+**	14	7	21
	**-**	6	5	11
**Total**		20	12	32
P = 1.000				

**Table 5:** Compaison of the Mycobacteria detected between lungs with lesions and lungs without lesions by PYRO.

		Lungs with lesion		Total
		+	-	
**Lungs without lesion**	**+**	11	7	18
	**-**	3	11	14
**Total**		14	18	32
P = 0.334				

## Discussion

In this study, we showed that a method based on a barcoded primer and 454 pyrosequencing provided profiles of microbiota samples from TB patients and healthy individuals. Although alveolar lavage fluid was the ideal carrier of representative information about the composition of the microbiota of patients’ lungs, we confronted a problem that requires further discussion here.

Although the possibility of a sterile state in the normal lower respiratory tract (LRT) has been negated recently, it was still difficult to obtain PCR-enriched production of microbial DNA from the alveolar lavage fluid because of the low quantities of LRT-colonising bacteria [23]. When compared with the sequencing reads from sputum samples in our previous study, the number of reads from the lavage fluid was significantly lower in this study. For this reason, we inevitably encountered the issue of whether the low number of reads was representative for profiling the microbiota. A rarefaction curve and Shannon-Weaver index were generated to evaluate the ability of the detected amplicons to reflect bacterial diversity in samples. Fig 1 indicates that almost all of the curves representing the samples consisted of two components: sharp slopes that were featured first, followed by decreased slopes in the second component. The shape and detailed data of the curves proved that the amplicons obtained were representative of the microbial diversity in the samples.

Charlson et al. proved the topographical continuity of bacterial populations in the human respiratory tract, indicating that the microbiota in the upper respiratory tract (URT) were significantly different from those in the lower respiratory tract (LRT) in quantity, but not in composition [23]. Therefore, we chose to use the secretions from the URT in healthy individuals as normal controls. According to our research, significant differences can be observed in the respiratory tract microbiota of healthy people when compared with TB patients. Fig 2 shows the phylogenetic relationship between the microbiota features of Groups H, A and B. Fig 2A shows that the green dots (Group H) and the blue/red dots (Groups A and B) were clearly located in different quadrants, indicating that the microbiota changed dramatically in TB patients. The distribution tendencies of these dots are also quite different. The green dots tended to cluster in a relatively small area; in contrast, dots representing Groups A and B had a relatively scattered clustering and were difficult to clearly separate, despite representing different microbiota origins. As Fig 2B shows, the microbiota from Group H concentrated under the same sub-branch and had a long phylogenic distance from Groups A and B.

These data suggest that healthy people had highly homogenous bacterial flora, while TB patients had a significantly altered bacterial community and a disappearance of any similarity. We interpreted the lack of homology as follows: from classical knowledge of TB pathology, *Mycobacteria* invasion is an important factor in injuring the mucosal barrier in the LRT, facilitating the colonisation of bacteria from the URT or foreign environments [24]. Our colleagues Cui et al. have found that many rare, unique bacteria exist in the microbiota of sputum from TB patients compared with that of healthy individuals, indicating its important role in creating the diversity in TB microbiota [25].

Fig 3 shows a hierarchical clustering heatmap that provides a visible pattern of the differences in microbiota. The abundance of each bacterial family in each patient is expressed by the colour ladder. The microbiota from Group H clustered in the right region of the figure, and their distribution differed significantly from that of the left region. The underlying reason for these differences was revealed when the data were analysed at the genera level.

Table 2 shows the top 30 genera with the highest abundance in Groups H and A/B. *Streptococcus* was the dominant genus in the bacterial community of healthy people; however, in the TB patients, the dominant genus was *Cupriavidus*. The percentage of *Streptococcus* decreased significantly in TB patients. According to previous knowledge, *Cupriavidus* seldom results in primary pulmonary infections in populations with normal immune function; however, it can cause secondary infections in patients with immune-compromising conditions, such as post-transplantation status, aplastic anaemia or cystic fibrosis [26,27,28]. As a chronic consuming disease, TB not only reduces systemic immunity but also damages the local physical barrier of the LRT; all of these factors contribute to colonisation by *Cupriavidus*. The bacterial flora from the LRT in TB patients was characterised by a high abundance of *Cupriavidus* compared with the characteristic *Streptococcus* found in healthy controls; this is the main reason why significant changes appeared in the microbiota of patients’ LRT. *Cupriavidus* was not found in healthy samples; as a result, we hypothesise that this genus is an important foreign infectious agent in TB patients.

Results from the pyrosequencing of patients found amplicons from more than 120 bacterial genera in alveolar lavage samples from both lungs. Comparisons of the microbiota from both lungs of each patient could clarify the change in the percentage of each genus in both sides. Table 3 shows the statistic calculations by Wilcoxon signed-rank tests to compare the abundance of each detected bacterial genera between Groups A and B. Other than *Mycobacteria* (P = 0.02) and *Porphyromonas* (P = 0.025), no genera showed statistically significant differences in abundance between the two sides (P>0.05). Table 5 compares the rate of *Mycobacteria* positive tests on both sides; there were no significant differences. These two tables suggest that despite wide distribution of *Mycobacteria* and *Porphyromonas* in both sides, the abundance in lesion-forming areas was much higher than in non-lesion forming areas. Thus, we hypothesise that these two genera were important lesion-forming factors. *Mycobacteria* are unquestionably the primary pathogens in TB and a main factor that stimulates macrophages to form lesions; however, we also consider *Porphyromonas* an important co-factor in lesion formation because of its high intra-lesion abundance. More research is needed to discover the underlying pathogenic mechanisms of *Porphyromonas*.

Last but not the least, the method that we used had its limit in identifying the amplicons’ belongings. The length of 16s rDNA V3 region amplicons were about 200–220 bp in length and by using the classification tool named RDP (Ribosomal Database Project), the amplicons could be precisely classified from phyla until genera level [21]. On the species level, the 200 bp amplicons could not be confirmed which species they belong to because the DNA chains were too short to provide enough information for identification. So, in our article, the change or the differences of microbiota were discussed on the genera level and the further researches are needed to disclose the details in the levels under the bacterial genera.

## Conclusions

In this study, we analysed the microbiota in lavage fluid to characterise the bacterial communities in TB patients’ LRT. Our results identified the following characteristics of TB microbiota. First, unlike the microbiota in healthy people, the dominant genus was *Cupriavidus* instead of *Streptococcus*, a main reason that the microbiota from TB patients differed from the healthy population. Second, *Cupriavidus* was the most important agent resulting in secondary and opportunistic infection in the context of TB. Finally, in addition to *Mycobacteria*, *Porphyromonas* may be a crucial agent because of its significantly increased proportion in lesions.

## Supporting Information

S1 File Scanning file of the pre-operation notification. This is the original manuscript in Chinese used in clinical work (in PDF format).
(PDF)

S2 File Scanning file of the translated pre-operation notification made by authorized translator. This is the translated file in English (in PDF format).
(PDF)
