# Supplementary material for: Correlation between Either Cupriavidus or Porphyromonas and Primary Pulmonary Tuberculosis Found by Analysing the Microbiota in Patients’ Bronchoalveolar Lavage Fluid
Source: PLoS One. 2015 May 22;10(5):e0124194. doi: 10.1371/journal.pone.0124194 (PMC4441454; doi:10.1371/journal.pone.0124194)
Supplement: S1 File — This is the original manuscript in Chinese used in clinical work (in PDF format). (PDF) [file pone.0124194.s001.pdf]

南 京 市 胸 科 医 院

纤维气管镜检查（治疗）知情同意书

患者姓名：孙永爱　　性别：男　　年龄：45岁　　病历号：144433

疾病介绍和治疗建议

医生已告知我患有 ：1.肺结核，需要在 局部麻醉下进行纤维气管镜检查（治疗）。

纤维支气管镜检查及常规镜下活检等对气管支气管及肺部疾病的明确诊断及治疗，有着非常重要的作用，纤维支气管镜经多年临床实践及广泛应用，已证实它有很高的安全性。但在检查或治疗过程中及其后，由于患者本身因素、病变部位及程度等原因，可能具有一定的危险性。

检查及治疗主要包括：

- ☑ 支气管镜检查
- ☑ 支气管镜下病变部位的活检或刷检及痰培养
- ☐ 气管、支气管异物的取出
- ☑ 某些疾病需要进行肺泡灌洗
- ☐ 支气管支架放置术和扩张术等
- ☐ 其他 —
- ☐ 气管、支气管息肉、肿瘤或闭塞的电凝、电切、钳除治疗

手术潜在风险和对策

医生告知我如下纤维气管镜检查术可发生的一些风险，有些不常见的风险可能没有在此列出，具体的手术术式根据不同病人的情况有所不同，医生告诉我可与我的医生讨论有关我手术的具体内容，如果我有特殊的问题可与我的医生讨论。

我理解任何手术麻醉都存在风险。

我理解任何所用药物都可能产生副作用，包括轻度的恶心、皮疹等症状到严重的过敏性休克，甚至危及生命。

我理解此手术可能出现的风险和医生的对策：

麻醉意外；肿瘤或病变难以切除放弃手术；

术中术后出血、休克、植物人，甚至死亡；术中损伤周围组织，重要神经、血管、脏器；

术中术后心脑血管意外，可致死亡；术中喉头、气管及支气管痉挛或窒息；术中返流或误吸；

术中出现气胸、血胸，导致脓胸、乳糜胸，需长期带管或再次手术；

肺炎、肺不张，急性呼吸窘迫综合征；术后局部皮肤麻木、疼痛、皮肤感觉消失；术后发热；

单侧喉返神经损伤，术后声音嘶哑，术后呛咳；双侧喉返神经损伤，术后呼吸困难、窒息；

急性肺栓塞；牙齿脱落、出血；置镜困难，终止手术；声带损伤，环杓关节脱位、下颌脱位；

术中严重缺氧、窒息；术中术后心律失常、心功能衰竭、心绞痛、心肌梗死；

支架损坏、移位，需再次手术取出或调整；术后支架刺激性咳嗽，需手术取出；术后肉芽组织生长或瘢痕狭窄，需再次手术处理；

术后仍存在严重的呼吸困难；其它难以预料的意外（如褥疮、泌尿系感染等）；除上述情况外，该患者行纤维气管镜检查术可能发生的其他并发症或者需要提请患者及家属特别注意的其他事项，如

—

南 京 市 胸 科 医 院

纤维气管镜检查（治疗）知情同意书

我理解如果我患有高血压、心脏病、糖尿病、肝肾功能不全、静脉血栓等疾病或者有吸烟史，以上这些风险可能会加大，或者在术中或术后出现相关的病情加重或心脑血管意外，甚至死亡。

我理解术中术后如果我的体位不当或不遵医嘱，可能影响手术效果。

特殊风险或主要高危因素

我理解根据我个人的病情，我可能出现未包括在上述所交待并发症以外的风险：

一旦发生上述风险和意外，医生会采取积极应对措施。

患者知情选择

我的医生已经告知我将要进行的手术方式、此次手术及术后可能发生的并发症和风险、可能存在的其它治疗方法并且解答了我关于此次手术的相关问题。

我同意在手术中医生可以根据我的病情对预定的手术方式做出调整。

我理解我的手术需要多位医生共同进行。我并未得到手术百分之百成功的许诺。

我授权医师对手术切除的病变器官、组织或标本进行处置，包括病理学检查、细胞学检查和医疗废物处理等。

患者签名________________ 签名日期 ____年 ____月____日

如果患者无法签署知情同意书，请其授权的亲属在此签名：

患者授权亲属签名__________ 与患者关系________ 签名日期____年___月____日

医生陈述

我已经告知患者将要进行的手术方式、此次手术及术后可能发生的并发症和风险、可能存在的其它治疗方法并且解答了患者关于此次手术的相关问题。

医生签名________________ 签名日期 ____年 ____月____日
