# Supplementary material for: Correlation between Either Cupriavidus or Porphyromonas and Primary Pulmonary Tuberculosis Found by Analysing the Microbiota in Patients’ Bronchoalveolar Lavage Fluid
Source: PLoS One. 2015 May 22;10(5):e0124194. doi: 10.1371/journal.pone.0124194 (PMC4441454; doi:10.1371/journal.pone.0124194)
Supplement: S2 File — This is the translated file in English (in PDF format). (PDF) [file pone.0124194.s002.pdf]

上海市外事翻译工作者协会
SHANGHAI INTERPRETERS' ASSOCIATION
上海市北京西路1277号1607室 邮编: 200040 电话、传真: 63239910, 62898328, 63233608, 63239181
Rm.1607, 1277 Beijing Rd.(W)Shanghai 200040 E-mail: fanyixiehui@vip.citiz.net http://www.shwsfy.com

Nanjing Chest Hospital

Informed Consent for Fiber Bronchoscopy(Treatment)

Patient name: Gender: Male/Female Age: MRN:

Introduction to disease and treatment recommendation:

The doctor has told me that I have pulmonary tuberculosis and need to receive fiber bronchoscopy(treatment) under local anesthesia. The fiber bronchoscopy, routine biopsy and others are important in the diagnosis and treatment of tracheo-bronchial and lung disease. After years of clinical practice and extensive application, fiber bronchoscopy proved to be of high security. But during or after examination and treatment, it still may have a certain degree of risk, because of patient' s individual factors, the site and extent of the lesion and other reasons.

The examination mainly includes:

☐ √ Brochoscopy

☐ √ Bronchoscopic lesion biopsy, or brush inspection and sputum culture

☐ Removal of foreign body from the trachea and bronchus.

☐ √ Alveolar wash is needed for certain disease.

☐ Bronchial stent placement surgery, dilatation and others

☐ Others.

☐ Electric coagulation, electricity cutting and forceps removal for Tracheo-bronchial polyps, tumor or obstruction.

Surgery potential risks and countermeasures:

The doctor has told me the possible risks of fiber bronchoscopy and that some uncommon risks may not be listed here. Specific surgery modes are different for the different conditions of the patients. The doctor has told me that I may discuss with my doctor about details in my operation. If I have any special questions, I may discuss with my doctor.

I understand that there exists risk in any operation anesthesia.

I understand that there may be side effects in the use of any drugs, including symptoms from mild nausea, skin rash to severe allergic shock, or even those endangering lives.

I understand the risk the operation has and the countermeasure the doctor takes.

Anesthetic accident; tumor or lesion is difficult to remove and the operation is given up.

During or after the operation, there occurs bleeding, shock, plant man or even death;

During the operation are damaged surrounding tissue, important nerves, blood ves[illegible]

SHANGHAI INTERPRETERS ASSOCIATION 上海外事翻译工作者协会 翻译专用章 (1) CERTIFIED TRANSLATION

organs;
During or after the operation, there occurs cardio-cerebrovascular accident, which may lead to death;
During the operation, there occurs spasm or suffocation in the larynx, trachea or bronchus;
During the operation there occurs regurgitation or aspiration;
During the operation, there occur pneumothorax, hemothorax, which lead to pyothorax, chylothorax, resulting in long term with tube or re-operation;
Pneumonia, pulmonary closure, acute respiratory stress syndrome;
After operation occur local skin numbness, pain and skin feeling loss;
After the operation occur fever, unilateral recurrent laryngeal nerve injury;
Post-operative hoarseness, post-operative choking cough; Bilateral recurrent laryngeal nerve injury, post-operative respiratory difficulty, choking; acute pulmonary embolism;
Tooth loss, bleeding; Difficulty in setting mirror, and termination of the operation;
Injury in vocal cord, arytenoid dislocation, jaw dislocation; Intra-operative hypoxia, asphyxia; Intra-operative and post-operative arrhythmias, heart failure, angina pectoris, and myocardial infarction;
Damage or displacement of stent, which has to be removed or adjusted by re-operation;
Post-operative irritable cough caused by stent, which has to be surgically removed;
Post-operative growth of granulation tissue or scar stenosis, which needs to be managed by re-operation;
After the operation still exists severe respiratory difficulty; other unexpected accidents (such as decubitus, urinary tract infection and others); Besides the above conditions, there may be other complications from fiber bronchoscopy and other matters that the patient and relatives should be specially concerned about,
I understand if I have hypertension, heart disease, diabetes, liver and kidney dysfunction, or venous thrombosis and other disease, or with smoking history, then I may have greater risks for the operation. And during or after the operation, there may occur aggravation of related condition, cardio-cerebrovascular accident or even death.
I understand that during or after the operation if my posture is inappropriate or I do not follow the doctor' s instruction, the surgical results may be affected.

Special risks or major risk factors
I understand that considering my condition I may have other risks than those mentioned above.
Once there occur the above risks and accidents, the doctors will take active countermeasures.

Patient informed choice
My doctor has told me about the operation mode which I will receive, the possible complications and risk which might occur during and after the operation, and the other possible treatments for my condition. My doctor has also answered my questi[illegible]

CERTIFIED TRANSLATION

in relation to this operation.
I agree that in the course of the operation the doctor can make adjustment to the scheduled operation mode according to my condition.
I understand that my operation needs to be done jointly by a few doctors. I did not get the promise that the operation will be an one hundred percent success.
I authorize my doctors to dispose the diseased organs, tissue or specimen excised in operation, including wastes from pathological exam, cytological exam and medical treatment.

Patient signature____________________ Signature date______________________

If Patient is unable to sign the informed consent, please authorize the relative in this signature.

Authorized relative signature______ Relationship_______ Signature date__________

Doctor' s statement

I have told Patient about the operation mode which Patient will receive, possible complications and risk which might occur during and after the operation, and the other possible treatments for Patient' s condition. I have also answered my questions in relation to this operation.

Doctor' s signature______________________ Signature__________________________

SHANGHAI INTERPRETERS ASSOCIATION
上海外事翻译工作者协会
翻译专用章
(1)
CERTIFIED TRANSLATION
